# Influence of the Angiotensin Converting Enzyme Insertion or Deletion Genetic Variant and Coronary Restenosis Risk: Evidence Based on 11,193 Subjects

**DOI:** 10.1371/journal.pone.0083415

**Published:** 2013-12-13

**Authors:** Yang Pan, Fang Wang, Qin Qiu, Ren Ding, Baolong Zhao, Hua Zhou

**Affiliations:** 1 Department of Cardiology, Baoshan Hospital of Integrated Traditional Chinese and Western Medicine, Shanghai, People’s Republic of China; 2 Department of Cardiology, Shanghai First People's Hospital, Shanghai Jiao Tong University School of Medicine, Shanghai, People’s Republic of China; Children's National Medical Center, Washington, United States of America

## Abstract

The insertion/deletion (I/D) polymorphism of the gene encoding angiotensin converting enzyme is a controversial risk factor for restenosis after percutaneous transluminal coronary angioplasties (PTCA) in patients. Genetic association studies can be problematic to reproduce due to insufficient power, phenotypic heterogeneity, population stratification, small effect of the variant and even publication biases. To derive a more precise estimation of the relationship as well as to quantify the between-study heterogeneity and potential bias, a meta-analysis including 11,193 patients from 33 published cohort studies was performed. In a combined analysis, the summary per-allele odds ratio for restenosis was 1.31 (95% CI: 1.08-1.58, P = 0.006), and 1.22 (95% CI: 0.95-1.56, P = 0.12), for PTCA-stent and PTCA-balloon, respectively. In the subgroup analysis by ethnicity, significantly increased restenosis risks after PTCA-stent were found in Asians for the polymorphism; whereas no significant associations were found among Caucasians. As for restenosis risks after PTCA-balloon, no evidence of any gene-disease association was obtained in the stratiﬁed analyses according to ethnicity and study size. In conclusion, this meta-analysis demonstrated that the DD homozygous of ACE I/D polymorphism was significantly associated with elevated restenosis susceptibility after PTCA-stent among Asian populations.

## Introduction

Coronary artery disease represents the most important cause of sudden cardiac death. Percutaneous coronary intervention (PCI) for unblocking a narrowed coronary artery is a widely used technique for treating patients with angina or an acute coronary event. Initially, PCI was performed only with balloon catheters, but technical advances made it possible to improve patient outcome by the placement of bare metal stents (BMS), or later, drug eluting stents (DES) at the site of blockage [1–3]. The utility of percutaneous transluminal coronary angioplasty (PTCA) is limited by a high incidence of restenosis which affects 30% to 40% of patients [4]. Restenosis after balloon angioplasty depends predominantly on elastic vessel recoil as opposed to in-stent restenosis, which depends mainly on neointimal growth [5,6]. Compared with restenosis after balloon angioplasty, less is known about the mechanisms of restenosis after intracoronary stent placement; it is likely due to a predominant proliferative model of restenosis [7] because stent diameter remains constant after placement and arterial remodeling cannot occur. In fact, analyses with ultrasounds have shown that restenosis after coronary stenting and after balloon PTCA differ in the amount of tissue proliferation [8], which is almost invariably observed within stents. Many potential risk factors for restenosis after angioplasty have been investigated including diabetes mellitus, age, hypertension, hyperlipidaemia [9-12]. However, none of these known risk factors for atherosclerosis or ischemic cardiovascular disease except for diabetes mellitus has been found to be associated with the occurrence of this complication [13]. As only 30% of restenosis could be predicted from clinical and angiographic variables [11], genetic factors are believed to be an important reason for inter-individual differences in treatment response [14,15]. 

 One ubiquitous system that may influence the restenosis process is the renin-angiotensins system (RAS). Angiotensin II is a potent vascular smooth muscle mitogen and may therefore play a pivotal role in the restenosis process [16]. Angiotensin I converting enzyme (ACE) is a core factor for the production of angiotensin II and the degradation of bradykinin [17]. High ACE levels have been reported to increase the risk of coronary thrombosis through the enhanced production of plasminogen activator inhibitor-I [18]. In addition, ACE may also interfere with coronary vasomotion [19]; high plasma ACE levels may lead to increased arterial wall thickness [20]. Moreover, experimental studies point towards the major role of the RAS in vessel healing after PTCA [21,22]. A common insertion/deletion (I/D) polymorphism in the gene encoding ACE has consistently been found associated with differential plasma ACE levels [23,24]. Furthermore, serum plasma activity of ACE has been thought to play a major role in the development of restenosis after coronary stent implantation [25].

 After the first report of an association between the I/D polymorphism and restenosis after PTCA [26], a number of studies have investigated the association between ACE I/D polymorphism and restenosis risk. However, the results were inconsistent or even contradictory. The lack of concordance across many of these studies reflects limitation in the studies, such as small sample size, ethnic difference, false positive results, and study design. With the increased studies in recent years among Asians and some other ethnic populations, there is a need to reconcile this inconsistency and to clarify the problems in previous studies. Therefore, we carried out a comprehensive meta-analysis on all eligible studies to estimate the overall restenosis risk of ACE I/D polymorphism as well as to quantify the between-study heterogeneity and potential bias.

## Materials and Methods

### Literature search strategy

Our analysis included all genetic association studies of the angiotensin converting enzyme insertion or deletion polymorphism in coronary restenosis after a percutaneous coronary intervention, with or without coronary stenting, conducted before the end of Sep. 2013. To accomplish this task, the MEDLINE, PubMed, ISI Web of Science, EMBASE, EBSCO, Cochrane Library databases, Wanfang and CNKI (China National Knowledge Infrastructure) databases were searched. For the electronic searches we used combinations of key words relating to the angiotensin converting enzyme gene (for example, angiotensin converting enzyme, ACE, peptidyl-dipeptidase A, polymorphism, variant, insertion/deletion, I/D, D/I) and to restenosis (for example, coronary, restenosis, percutaneous, angioplasty, PTCA, stent, stenting). To reduce the likelihood of publication bias, unpublished data, negative data from candidate gene studies, and GWAS (genome-wide association studies) data were also actively sought. Articles were selected on the basis of the abstract, before examining the full text. In addition, the reference lists of selected articles were hand-searched to identify additional relevant reports. Case reports, case-only studies, editorials, and review articles were excluded. Articles in languages other than English were translated. 

### Inclusion criteria and data extraction

An eligible study for inclusion was restricted to prospective cohort studies and case-control studies in which the relative risk (RR) or odds ratio (OR) of coronary restenosis in relation to the ACE I/D polymorphism was reported or exact figures were available that permitted estimation of the OR. Restenosis was defined as a narrowing of the coronary diameter by 50% or more at follow up compared with the minimal luminal diameter immediately after intervention. Only articles that actually did report angiographic follow-up data were included in the analysis. Because restenosis is a time-related phenomenon, a follow-up window from 3 to 12 months was chosen [27,28]. If more than one article was published using the same case series, only the study with the largest sample size was included. 

Two investigators extracted information from all eligible publications independently according to the inclusion criteria listed above. Data were collected on the authors, journal, year of publication, ethnicity of participants, studies design (prospective cohort study or case-control study), coronary intervention procedure, genotype distribution in the case and control group for II, I/D, and DD genotypes, consistency of genotype frequencies with Hardy-Weinberg equilibrium (HWE), restenosis definition criteria, age, sex, duration of follow up and genotyping methods. Disagreements were resolved by discussion among all authors.

### Quality assessment: extended-quality score

For association studies with inconsistent results on the same polymorphisms, the methodological quality should be assessed by appropriate criteria to limit the risk of introducing bias into meta-analyses or systematic reviews. A procedure known as ‘extended-quality score’, has been developed to assess the quality of association studies. The procedure scores each paper categorizing it as having ‘high’, ‘median’ or ‘poor’ quality. Detailed procedure of the quality assessment was previously described [29].

### Data synthesis and analysis

The OR for restenosis risk associated with the ACE I/D variant was assessed in each study, along with its 95% confidence interval (CI). The meta-analysis examined the association between the polymorphism and the risk of restenosis, for the: (i) allele contrast, (ii) heterozygous contrast, (iii) homozygote contrast, (iv) dominant, and (v) recessive models [30]. Genotype counts or allelic counts for cases and controls from each original study were used to estimate summary ORs. We did not use adjusted ORs to estimate summary ORs since inconsistent covariates were used for adjustment in original studies included in this meta-analysis. The between-study heterogeneity was explored by the Cochran χ^2^ based Q test, and the quantity of heterogeneity was assessed by the inconsistency index I^2^ statistic (ranging from 0 to 100%), which is deﬁned as the percentage of the observed between-study variability that is due to heterogeneity rather than chance [31,32]. Heterogeneity was considered signiﬁcant if P<0.10. Generally, I^2^ values <25% correspond to no or little heterogeneity, values 25–50% correspond to moderate heterogeneity, and values >50% correspond to strong heterogeneity between studies. The pooled OR was estimated using random effects (RE) models [33]. RE modeling assumes a genuine diversity in the results of various studies, and it incorporates to the calculations a between-study variance. When there is lack of heterogeneity, the RE model coincides with the ﬁxed effects model [34]. The significance of the overall OR was determined by the Z-test. 

Subgroup analysis was stratiﬁed by the study characteristic of ethnicity (Asian vs. Caucasian), and study size, respectively. Ethnic group, study size, duration of follow up, age and sex were analyzed as covariates in meta-regression. A cumulative meta-analysis was performed to evaluate the trend of OR in time [35]. In cumulative meta-analysis, studies were chronologically ordered by publication year, then the pooled ORs were obtained at the end of each year, i.e., at each information step. Cumulative meta-analysis provides a frame work for updating a genetic effect from all studies as evidence accumulates [35]. To evaluate the stability of the results, we performed a sensitivity analysis by removing one study at a time. The potential publication bias was explored for both the OR and the standardized mean difference by visual interpretation of the funnel plot [36], and the asymmetry of the funnel plot was checked with Egger’s test [37]. All analyses were done using STATA software, version 11.0 (STATA Corp., College Station, TX, USA). All P-values were two tailed. This meta-analysis is guided by the PRISMA statement (Checklist S1).

## Results

### Study Characteristics

Based on our search strategy, the primary screening produced 221 potentially relevant articles. 188 articles were excluded because they clearly did not meet the inclusion criteria or overlapping references. Study selection process was shown in Figure S1. Finally, a total of 33 Cohort studies with 11,193 patients investigating restenosis were included [20,38-69]. There are 23 data sets from 21 studies with 1,952 restenosis cases and 6,598 controls concerning bare-metal stent deployment (PTCA-stent) and 12 data sets from 12 studies involving 1,076 restenosis cases and 1,567 controls concerning balloon angioplasty (PTCA-balloon). The extended-quality scores ranged from 5 to 8, and 12 studies were given median quality, whereas 21 were given high quality. No “poor quality” study was found. The main characteristics of these studies are described in Table 1.

**Table 1:** Characteristics of studies included in a meta-analysis.

Reference	Year	Ethnicity	Duration of follow up	Intervention type	Definition of restenosis cases	No. of cases/controls	Mean age of patients	Gender distribution in patients (male %)	Study quality
Ohishi [26]	1993	Japanese	6 months	PTCA-balloon	diameter stenosis >50%	32/50	NA	NA	High
Beohar [38]	1995	American	3 months	PTCA-balloon	diameter stenosis ≥50%	64/25	63.9	NA	High
van Bockxmeer [39]	1995	Australian	6 months	PTCA-balloon	diameter stenosis >50%	88/119	57.0	82.1	High
Kamitani [40]	1995	Japanese	6 months	PTCA-balloon	diameter stenosis >50%	38/52	52.0	100	High
Kaski [41]	1996	Spanish	6 months	PTCA-balloon	diameter stenosis ≥50%	35/34	58.0	82.6	Median
Tsukada [42]	1997	Japanese	3 months	PTCA-balloon	diameter stenosis ≥50%	25/71	60.0	NA	Median
Hamon [43]	1998	French	6 months	PTCA-balloon	diameter stenosis >50%	116/155	60.0	84.5	High
Yoshida [44]	1999	Japanese	5.2 years	PTCA-balloon	diameter stenosis ≥50%	47/123	58.2	NA	High
Okamura [45]	1999	Japanese	6 months	PTCA-balloon	diameter stenosis >50%	19/27	60.0	86.6	High
Völzke [46]	2000	German	6 months	PTCA-balloon	diameter stenosis >50%	160/351	60.6	75.9	High
Zee [47]	2001	Spanish	6 months	PTCA-balloon	diameter stenosis >50%	342/437	58.9	89.2	High
Samani [48]	1995	British	4 months	PTCA-balloon	diameter stenosis ≥50%	110/123	NA	83.3	High
Amant [49]	1997	French	6 months	PTCA-stent	diameter stenosis >50%	59/99	60.0	80.1	High
Gensini [50]	1999	Italian	6 months	PTCA-stent	diameter stenosis ≥50%	27/130	NA	NA	Median
Guarda [51]	1999	Chilean	6 months	PTCA-stent	diameter stenosis >50%	22/26	NA	NA	Median
Gürlek [52]	2000	Turkish	6 months	PTCA-stent	diameter stenosis ≥50%	51/107	53.0	84.8	High
Koch [53]	2000	German	12 months	PTCA-stent	diameter stenosis ≥50%	513/1043	62.9	78.8	High
Jørgensen [54]	2001	Dane	6 months	PTCA-stent	diameter stenosis >50%	49/320	59.0	79.4	High
Taniguchi [55]	2001	Japanese	6 months	PTCA-stent	diameter stenosis >50%	26/41	65.2	74.6	Median
Ferrari [56]	2002	German	6 months	PTCA-stent	diameter stenosis ≥50%	39/115	61.0	77.3	High
Ryu [57]	2002	Korean	6 months	PTCA-stent	diameter stenosis >50%	64/191	59.5	74.8	High
Gomma [58]	2002	British	6 months	PTCA-stent	diameter stenosis ≥50%	60/144	59.4	75.6	High
Qu [59]	2002	Chinese	3 months	PTCA-stent	diameter stenosis ≥50%	43/85	68.0	84.4	Median
Okumura [60]	2002	Japanese	6 months	PTCA-stent	diameter stenosis ≥50%	16/76	64.3	79.3	Median
Ribichini [61]	2003	Italian	6.3 months	PTCA-stent	diameter stenosis ≥50%	271/727	61.0	82.2	High
Guo [62]	2005	Chinese	6 months	PTCA-stent	diameter stenosis ≥50%	30/73	70.0	NA	Median
Wang [63]	2005	Chinese	6 months	PTCA-stent	diameter stenosis ≥50%	62/40	62.0	88.2	Median
Guneri [64]	2005	Turkish	9 months	PTCA-stent	diameter stenosis ≥70%	48/48	59.6	62.8	High
Wang [65]	2005	Chinese	6 months	PTCA-stent	diameter stenosis ≥50%	58/139	NA	NA	Median
Ujiie [66]	2006	Japanese	7 months	PTCA-stent	diameter stenosis >50%	15/60	66.9	78.6	Median
Gao [67]	2006	Chinese	6 months	PTCA-stent	diameter stenosis ≥50%	102/247	NA	NA	Median
Wijpkema [68]	2006	Dutch	9 months	PTCA-stent	diameter stenosis >50%	316/2572	62.0	70.9	High
Lv [69]	2012	Chinese	6 months	PTCA-stent	diameter stenosis ≥50%	81/315	58.8	89.4	High

NA: not available

### ACE I/D polymorphism and restenosis risk after PTCA-stent

**Figure 1:**
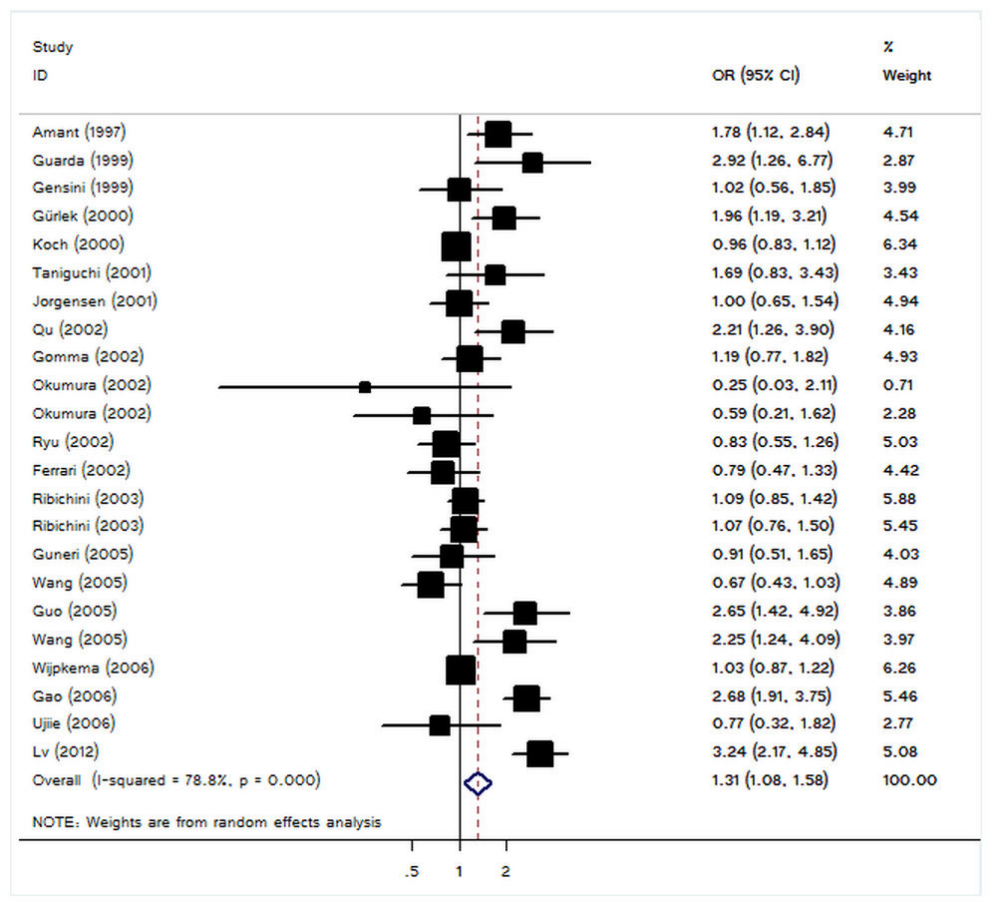
Forest plot from meta-analysis of ACE I/D polymorphism and restenosis risk after PTCA-stent.

For restenosis risk after PTCA-stent and I/D polymorphism of ACE, our meta-analysis gave an overall per-allele OR of 1.31 (95% CI: 1.08–1.58; P=0.006) with statistically signiﬁcant between-study heterogeneity (P<10^-4^) (Figure 1 and Table 2). Signiﬁcant associations were also detected for DD homozygous with OR of 1.59 (95% CI: 1.11-2.29, P=0.01, Table 2); similar results still maintained using recessive genetic model (OR=1.60, 95% CI: 1.20-2.13, P=0.001). However, no significant association was detected for ID heterozygous (Table 2). In view of significant heterogeneity and to seek for its potential sources, we performed a panel of subgroup analyses on ethnicity and study size. When studies were stratiﬁed for ethnicity, signiﬁcant risks were found among Asians for DD genotype (DD homozygote: OR = 2.18, 95%CI: 1.08-4.40, P=0.03; recessive model: OR = 1.79, 95%CI: 1.10-2.92, P=0.02).However, we failed to detect any association to restenosis risk for Caucasians in all genetic models. In considering sample size subgroups, the per-allele OR was 1.27 (95% CI: 0.98-1.65, P =0.07) in small studies compared to 1.36 (95% CI: 1.01-1.85, P =0.046) in larger studies (Table 2). 

**Table 2:** Results of meta-analysis for ACE I/D polymorphism and restenosis risk after PTCA-stent.

Genetic model	Overall association (23 datasets)		Subgroup analysis by ethnicity				Subgroup analysis by study size			
			Caucasian (10 datasets)		Asian (13 datasets)		Large (7 datasets)		Small (16 datasets)	
ACE I/D polymorphism	ORs (95% CI); P(Z)	Test for heterogeneity P(Q); I^2^	ORs (95% CI); P(Z)	Test for heterogeneity P(Q); I^2^	ORs (95% CI); P(Z)	Test for heterogeneity P(Q); I^2^	ORs (95% CI); P(Z)	Test for heterogeneity P(Q); I^2^	ORs (95% CI); P(Z)	Test for heterogeneity P(Q); I^2^
D allele	1.31 (1.08-1.58); 0.006	<10^-4^; 79%	1.08 (0.95-1.22); 0.25	0.14; 34%	1.44 (1.00-2.08); 0.048	<10^-4^; 81%	1.36 (1.01-1.85); 0.046	<10^-4^; 90%	1.27 (0.98-1.65); 0.07	<10^-4^; 67%
Dominant model	1.19 (0.93-1.52); 0.16	<10^-4^; 63%	0.99 (0.85-1.15); 0.89	0.47; 0%	1.61 (1.00-2.58); 0.05	<10^-4^; 71%	1.28 (0.88-1.86); 0.19	<10^-4^; 78%	1.14 (0.80-1.62); 0.47	0.007; 53%
Recessive model	1.60 (1.20-2.13); 0.001	<10^-4^; 75%	1.33 (0.99-1.78); 0.06	0.001; 67%	1.79 (1.10-2.92); 0.02	<10^-4^; 72%	1.57 (1.01-2.45); 0.04	<10^-4^; 88%	1.62 (1.10-2.38); 0.01	0.001; 60%
Heterozygous	1.03 (0.82-1.30); 0.80	0.004; 50%	0.85 (0.66-1.09); 0.19	0.08; 41%	1.41 (0.95-2.09); 0.09	0.02; 50%	1.12 (0.83-1.51); 0.45	0.01; 62%	0.97 (0.68-1.38); 0.87	0.04; 43%
Homozygous	1.59 (1.11-2.29); 0.01	<10^-4^; 74%	1.04 (0.82-1.32); 0.75	0.22; 24%	2.18 (1.08-4.40); 0.03	<10^-4^; 77%	1.69 (0.94-3.03); 0.08	<10^-4^; 87%	1.54 (0.94-2.52); 0.08	<10^-4^; 61%

P(Z): Z test used to determine the signiﬁcance of the overall OR.

P(Q): Cochran’s chi-square Q statistic test used to assess the heterogeneity in subgroups.

Meta-regression was used to explore the cause of heterogeneity, and it was found that follow up (P = 0.07), mean age of cases (P = 0.11), sex distribution (P = 0.19), did not significantly correlated with the magnitude of the genetic effect. However, a slight effect in heterogeneity for ethnicity (P = 0.02) and sample size (P = 0.007) were found. 

Angiotensin converting enzyme inhibitors have been evaluated as prophylactic treatment against restenosis in several studies, but the relationship between the ACE I/D polymorphism and ACE inhibitors using in modifying restenosis risk was not established yet. Genotype counts of the ACE I/D polymorphism among patients stratiﬁed by ACE inhibitors treatment were available in 5 studies including 438 restenosis cases after PTCA-stent. Patients on ACE inhibitor treatment with the DD genotype of the ACE gene had similar in-stent restenosis risk compared with those patients without this treatment with an OR of 1.25 [95% CI: 0.82-1.89, P(Z)=0.30, P(Q)=0.48, I^2^=0%; Figure S2] 

 Retrospective analysis based on the publication year showed that the cumulative results (asymptote lines) tended to be stable for the eight studies after 2002, but more replications would be necessary for complete conﬁrmation (Figure S3).

 Sensitivity analysis was performed by excluding one study at a time. The results confirmed the significant association between the DD homozygous and the risk of restenosis, with ORs and 95% CIs ranging from 1.42 (95% CI: 1.02-1.12) to 1.67 (95%CI: 1.11-2.50). Funnel plot and Egger’s test were performed to evaluate the publication bias of the literature reviewed. The shape of the funnel plots seemed symmetrical, suggesting no publication bias among the studies included (Figure S4). The Egger test (data not shown) provided further evidence that there was no publication bias among the studies included (P =0.11).

### ACE I/D polymorphism and restenosis risk after PTCA-balloon

**Figure 2:**
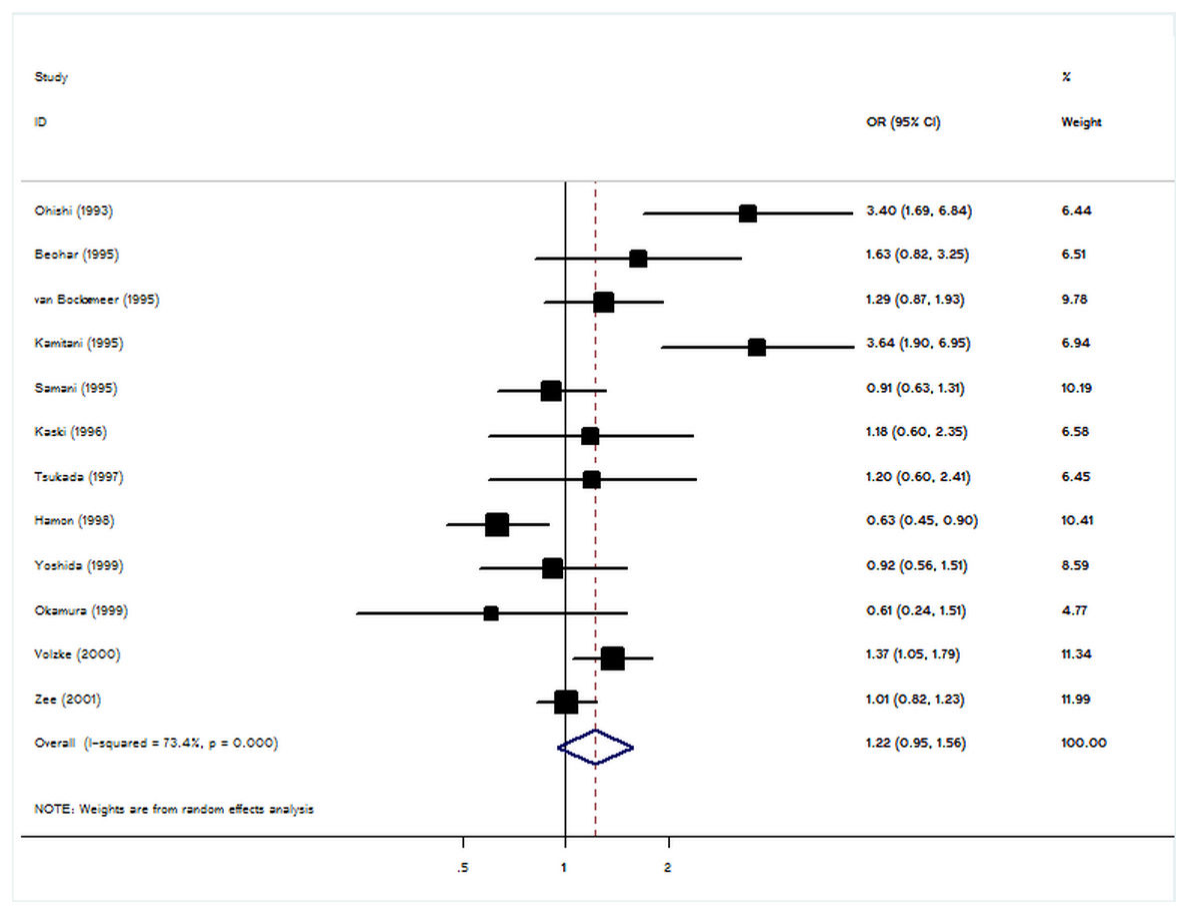
Forest plot from meta-analysis of ACE I/D polymorphism and restenosis risk after PTCA-balloon.

Significant heterogeneity was present among the 12 datasets of the ACE I/D polymorphism (Table 3). In meta-regression analysis, study size (P = 0.02) was associated with lnOR, thus explained a large part of the heterogeneity, whereas ethnicity (P = 0.16), mean age of patients (P = 0.73), duration of follow up (P=0.23), and sex distribution of cases (P = 0.38) explained little heterogeneity. In the overall analysis, the risk D variant was not signiﬁcantly associated with elevated restenosis risk after PTCA-balloon (D allele: OR= 1.22, 95% CI: 0.95-1.56, P=0.12; Figure 2 and Table 3). No signiﬁcant associations were found for heterozygous or homozygous of the polymorphism SNP which in line with the results under dominant or recessive genetic models (Table 3). In addition, we still did not observe an association between ACE genotype and restenosis risk in the stratiﬁed analysis according to ethnicity or study size (Table 3).

**Table 3:** Results of meta-analysis for ACE I/D polymorphism and restenosis risk after PTCA-balloon.

Genetic model	Overall association (12 datasets)		Subgroup analysis by ethnicity				Subgroup analysis by study size			
			Caucasian (7 datasets)		Asian (5 datasets)		Large (2 datasets)		Small (10 datasets)	
ACE I/D polymorphism	ORs (95% CI); P(Z)	Test for heterogeneity P(Q); I^2^	ORs (95% CI); P(Z)	Test for heterogeneity P(Q); I^2^	ORs (95% CI); P(Z)	Test for heterogeneity P(Q); I^2^	ORs (95% CI); P(Z)	Test for heterogeneity P(Q); I^2^	ORs (95% CI); P(Z)	Test for heterogeneity P(Q); I^2^
D allele	1.22 (0.95-1.56); 0.12	<10^-4^; 73%	1.06 (0.85-1.32); 0.60	0.02; 61%	1.55 (0.79-3.06); 0.60	0.02; 61%	1.16 (0.86-1.57); 0.33	0.07; 69%	1.25 (0.88-1.78); 0.21	<10^-4^; 76%
Dominant model	1.14 (0.71-1.84); 0.58	<10^-4^; 78%	0.94 (0.48-1.81); 0.84	<10^-4^; 85%	1.55 (0.81-2.97); 0.18	0.08; 53%	1.30 (0.84-2.01); 0.24	0.15; 52%	1.12 (0.58-2.17); 0.74	<10^-4^; 81%
Recessive model	1.35 (1.00-1.79); 0.05	0.03; 48%	1.16 (0.96-1.40); 0.12	0.70; 0%	1.64 (0.63-4.29); 0.31	0.02; 67%	1.17 (0.78-1.75); 0.45	0.11; 60%	1.43 (0.98-2.09); 0.06	0.05; 47%
Heterozygous	0.99 (0.60-1.62); 0.95	<10^-4^; 77%	0.85 (0.40-1.80); 0.67	<10^-4^; 87%	1.23 (0.78-1.94); 0.37	0.79; 0%	1.24 (0.90-1.71); 0.19	0.30; 6%	0.93 (0.46-1.87); 0.84	<10^-4^; 80%
Homozygous	1.28 (0.75-2.16); 0.36	<10^-4^; 73%	1.06 (0.58-1.91); 0.86	<10^-4^; 76%	1.78 (0.58-5.53); 0.31	0.02; 67%	1.40 (0.74-2.67); 0.30	0.06; 71%	1.25 (0.59-2.62); 0.56	<10^-4^; 76%

P(Z): Z test used to determine the signiﬁcance of the overall OR. P(Q): Cochran’s chi-square Q statistic test used to assess the heterogeneity in subgroups.

Although cumulative meta-analysis for I/D polymorphism in restenosis indicated a downward trend of association in the whole studied period (1993–2001; Figure S5), it is evident that this trend is attributed to the ﬁrst four studies.

In sensitivity analysis, statistically similar results were obtained after sequentially excluding each study, indicated that no single study influenced the pooled OR qualitatively, suggesting that the results of this meta-analysis are stable. The shape of the funnel plots was symmetrical (Figure S6). The statistical results still did not show publication bias in these studies (P=0.80).

## Discussion

Restenosis after PCI is an important clinical problem and is a response to injury of the vessel wall, platelet aggregation, thrombus formation, liberation of growth factors, cellular hyperplasia involving predominantly smooth muscle proliferation and migration, and intercellular matrix formation [4,39,49,70,71]. So far, the etiological basis of restenosis is only partly understood. The injury induced by PCI within the vascular wall causes segmental thrombus formation and subsequent invasion of macrophages and polymorphonuclear leukocytes in the blood vessel. This process is followed by release of numerous growth factors from blood cells and stretched smooth muscle cells that lead to the proliferation of smooth muscle cells in the treated lesion [72,73]. In the past decade, a number of studies have been carried out to investigate the relationship between the ACE I/D polymorphism and restenosis risk after PTCA [45-49] since it was first reported to affect restenosis rate in a small number of Japanese patients [26]. However, these studies have yielded contradictory results. In the current meta-analysis, on the basis of 33 cohort studies involving 11,193 patients, we quantitatively assessed the effect of ACE I/D variant and restenosis risk after PTCA. 

Through the combined examination of independent studies, we did not ﬁnd evidence supporting a positive relation between restenosis risk after PTCA-stent and I/D polymorphism of ACE. For the subgroup analysis based on ethnicity and study size, we were unable to observe any effect modiﬁcation in most comparison, which is in line with the pooled analysis. However, significant associations were found in Asians but not for Caucasians when stratified by ethnicity, suggesting a possible role of ethnic differences in genetic backgrounds and the environment they lived in. In fact, the distribution of the less common D allele varies extensively between different races, with a prevalence of 37% among Caucasians [47], and 55% among Asians [69]. Thus, failing to identify any significant association in Caucasian populations could be due to substantially lower statistical power caused by the relatively lower prevalence of D allele. Therefore, additional studies are warranted to further validate ethnic difference in the effect of this functional polymorphism on restenosis risk. Such result could also be due to the limited number of studies among Caucasian populations, which had insufficient statistical power to detect a slight effect or different linkage disequilibrium pattern of the polymorphism among these populations. Furthermore, study design or small sample size or some environmental factors may affect the results. As post-PTCA restenosis is a complex process, it is possible that variation at this locus has modest effects on restenosis, but environmental factors (i.e. diabetes, hypercholesterolemia, hypertension, smoking, obesity) may predominate in the progress of restenosis, and mask the effects of this variation. Moreover, clinical heterogeneity like age, dietary, years from onset and disease severity may also explain the discrepancy. Since the Asian population reports in the subgroup analysis include a mixture of populations from very distant countries, the result must be interpreted with caution for significant between-study heterogeneity identified. 

 It is reported that the level of plasma ACE is stable in an individual, but it is highly variable across individuals [74]. Previous studies reported that plasma ACE and cardiac ACE activity were elevated in individuals with DD genotype [74,75]. This increased ACE activity may due partly to the higher degree of neointimal thickening observed in D allele carriers. Bonithon-Kopp et al. found that chronic exposure to high levels of plasma ACE resulted in vascular wall thickening [20]. Castellano et al. also reported that ACE DD genotype may be a risk factor for the development of common carotid intima-media thickening [76]. These studies suggested that high levels of plasma ACE may causes hypertrophic changes of the vessel wall, thus lead to the failure of PTCA-stent. 

 Our results demonstrated that the D variant of I/D polymorphism on ACE is not a risk factor for developing restenosis after PTCA-balloon. In addition, the meta-analyses for the risk of restenosis following stent angioplasty and treatment with ACE inhibitors produced non-significant results. However, the subgroup meta-analyses considering interactions between ACE genotype and ACE inhibitors was performed on the basis of a fraction of all the possible data to be pooled, so selection bias may have occurred and our results may be over inflated. In this context, more reliable results can be expected if individual data are available for a pooled analysis. Thus, the result must be interpreted with caution. Besides, the vast majority of subjects in the study are of PTCA-stent, and statistical power for analyses in PTCA-balloon is limited which may make it hard to detect a small effect. Evidence that ACE inhibitors effectively limit restenosis has been reported in animal models [77,78], but has proven ineffective for restenosis in humans after coronary balloon angioplasty in multicenter, double-blind, place-controlled trials [79-81]. Potential mechanisms by which ACE inhibition reduces neointimal hyperplasia in these models may be related to the role of this enzyme in the formation of angiotensin II, a potent growth factor for smooth muscle cells [82], and in the degradation of bradykinin, a growth inhibitor for smooth muscle cells [83]. The implication of ACE in neointimal hyperplasia has been further supported by gene transfer studies showing that overexpression of the ACE gene increased DNA synthesis in the rat carotid artery [84]. Furthermore, experimental [85,86] and clinical studies have suggested that the contribution of neointimal hyperplasia to restenosis after balloon angioplasty is relatively limited and that lumen renarrowing is in fact related primarily to vessel remodeling (ie, chronic sclerosis with vessel constriction) [87]. Conversely, because the stent prevents the remodeling process, restenosis after coronary stenting is primarily a consequence of neointimal hyperplasia within the stent [6]. Thus, factors that directly affect the degree of neointimal hyperplasia will be more likely to influence restenosis after coronary stenting than restenosis after balloon angioplasty.

In the meta-analysis, only the unadjusted pooled ORs were calculated, because data for possible confounding factors that influence the estimates of associations (e.g., age, sex, types of stent or balloon implanted, ACE inhibitors treatment, and life-style) were not provided. Ideally we would like to pool individual-level data. However this is not possible for the present study. Sampling variability and stratiﬁcation in genetic association studies could be a possible confounding factor on the role of genetic markers. The strict selection criteria ensure a clear case and control deﬁnition for meta-analysis, because when the possibility for a case to be considered as a control is minimized, then the estimation of risk is unbiased. The cases and controls of each study were well deﬁned with similar inclusion criteria, although they unavoidably cover a wide spectrum of disease, in terms of duration, demographics, and other clinical manifestations. The existence of diversity of these factors across studies may result to the presence of heterogeneity. In addition, the risk effect may depend on the interaction with other risk factors: smoking, alcohol consumption, exercise, control of diabetes, and body mass index, all of which modulate the development of restenosis [88,89]. Prevalence of restenosis depends on age, and it is maximized in elderly individuals. Thus, the absence of restenosis in young patients does not exclude the possibility of developing restenosis later. In many studies, younger individuals were frequently included as controls. Therefore, if a control group may include cases that are still at risk for developing restenosis, then there is a fundamental risk of bias in these studies.

 Limited statistical power is a common problem in complex genetic studies. In this meta-analysis, to obtain as much literature as possible, we put equal emphasis on the positive and negative literature, which reduced potential publication bias and helped to maximize statistical power and robustness. Documentation of the nature of the study group and full angiographic follow-up are also essential in any study on restenosis. When restenosis is described as a binary variable, it is rational to incorporate a measure of the loss of the gain attained by PTCA or the loss of lumen from the time of PTCA into a discrete criterion rather than using 50% of lumen diameter as a single criterion. The influence of genetic polymorphisms in the development of restenosis has been investigated by means of candidate gene approaches. Spectacular advance was made in identifying susceptible genes involved in various types of diseases through genome-wide association strategy (GWAS). In the future, GWAS may be of great importance when establishing a comprehensive picture of the relationship between genetic variant and post-PTCA outcome.

 In summary, findings from this meta-analysis indicate that the DD homozygous of ACE I/D polymorphism was significantly associated with increased risk of restenosis after PTCA-stent among Asian populations. More work is needed to further investigate the association of the polymorphism across different ethnic populations. Besides, future studies are recommended to identify the possible gene-gene and gene-environmental interactions. 

## Supporting Information

Checklist S1 **PRISMA 2009 Checklist.**
(DOC)

Figure S1 **Flow chart of literature search for studies examining ACE I/D polymorphism and risk of restenosis after PTCA.**
(TIF)

Figure S2 **Association between ACE I/D polymorphism and restenosis risk after PTCA-stent by ACE inhibitors treatment.**
(TIF)

Figure S3 **Cumulative meta-analysis for restenosis after PTCA-stent and ACE I/D polymorphism: the random effects pooled odds ratio (OR) with the corresponding 95% confidence interval (CI) at the end of each year-information step is shown.**
(TIF)

Figure S4 **Begg’s funnel plot for publication bias in studies on ACE I/D polymorphism and restenosis after PTCA-stent.**
(TIF)

Figure S5 **Cumulative meta-analysis for restenosis after PTCA-balloon and ACE I/D polymorphism.**
(TIF)

Figure S6 **Begg’s funnel plot for publication bias in studies on ACE I/D polymorphism and restenosis after PTCA-balloon.**
(TIF)

## References

[1] Agema WR, Monraats PS, Zwinderman AH, De Winter RJ, Tio RA et al. (2004) Current PTCA practice and clinical outcomes in The Netherlands: the real world in the pre-drug-eluting stent era. Eur Heart J 25: 1163–1170. doi:10.1016/j.ehj.2004.05.006. PubMed: 15231375. 15231375

[2] Roiron C, Sanchez P, Bouzamondo A, Lechat P, Montalescot G (2006) Drug eluting stents: an updated meta-analysis of randomized controlled trials. Heart 92: 641–649. doi:10.1136/hrt.2005.061622. PubMed: 16216853. 16216853 PMC1860942

[3] Sigwart U, Puel J, Mirkovitch V, Joffre F, Kappenberger L (1987) Intravascular stents to prevent occlusion and restenosis after transluminal angioplasty. N Engl J Med 316: 701-706. doi:10.1056/NEJM198703193161201. PubMed: 2950322. 2950322

[4] Serruys PW, de Jaegere P, Kiemeneij F, Macaya C, Rutsch W et al. (1994) A Comparison of Balloon-Expandable-Stent Implantation with Balloon Angioplasty in Patients with Coronary Artery Disease.. N Engl J Med 331: 489-495. doi:10.1056/NEJM199408253310801. PubMed: 8041413. 8041413

[5] Andersen HR, Maeng M, Thorwest M, Falk E (1996) Remodelling rather than neointimal formation explains luminal narrowing after deep vessel injury: insights from a porcine coronary (re)stenosis model. Circulation 93: 1716 –1724. doi:10.1161/01.CIR.93.9.1716. PubMed: 8653878. 8653878

[6] Hoffmann R, Mintz GS, Dussaillant GR, Popma JJ, Pichard AD et al. (1996) Patterns and mechanisms of in-stent restenosis: a serial intravascular ultrasound study. Circulation 94: 1247–1254. doi:10.1161/01.CIR.94.6.1247. PubMed: 8822976. 8822976

[7] Mintz GS, Popma JJ, Pichard AD, Kent KM, Satler LF et al. (1996) Arterial remodeling after coronary angioplasty: a serial intravascular ultrasound study. Circulation 94: 35–43. doi:10.1161/01.CIR.94.1.35. PubMed: 8964115. 8964115

[8] Mintz GS, Popma JJ, Hong MK, Pichard AD, Kent KM et al. (1996) Intravascular ultrasound to discern device-specific effects and mechanism of restenosis. Am J Cardiol 78(suppl 3A): 18–22. doi:10.1016/S0002-9149(96)00493-6. PubMed: 8751842. 8751842

[9] Weintraub WS, Kosinski AS, Brown CL 3rd, King SB 3rd (1993) Can restenosis after coronary angioplasty be predicted from clinical variables? J Am Coll Cardiol 21: 6-14. doi:10.1016/0735-1097(93)90711-9. PubMed: 8417077. 8417077

[10] Violaris AG, Melkert R, Serruys PW (1995) Long-term luminal renarrowing after successful elective coronary angioplasty of total occlusions. A quantitative angiographic analysis. Circulation 91: 2140-2150. doi:10.1161/01.CIR.91.8.2140. PubMed: 7697842. 7697842

[11] Faxon DP (1995) Effect of high dose angiotensin-converting enzyme inhibition on restenosis: final results of the MARCATOR Study, a multicenter, double-blind, placebo-controlled trial of cilazapril. The Multicenter American Research Trial With Cilazapril After Angioplasty to Prevent Transluminal Coronary Obstruction and Restenosis (MARCATOR) Study Group. J Am Coll Cardiol 25: 362-369. doi:10.1016/0735-1097(95)92960-D. PubMed: 7829789. 7829789

[12] Peters RJ, Kok WE, Di Mario C, Serruys PW, Bär FW et al. (1997) Prediction of restenosis after coronary balloon angioplasty. Results of PICTURE (Post-IntraCoronary Treatment Ultrasound Result Evaluation), a prospective multicenter intracoronary ultrasound imaging study. Circulation 95: 2254-2261. doi:10.1161/01.CIR.95.9.2254. PubMed: 9142002. 9142002

[13] Van Belle E, Bauters C, Hubert E, Bodart JC, Abolmaali K et al. (1997) Restenosis rates in diabetic patients: a comparison of coronary stenting and balloon angioplasty in native coronary vessels. Circulation 96: 1454 –1460. doi:10.1161/01.CIR.96.5.1454. PubMed: 9315531. 9315531

[14] Arnett DK, Baird AE, Barkley RA, Basson CT, Boerwinkle E et al. (2007) Relevance of genetics and genomics for prevention and treatment of cardiovascular disease: a scientific statement from the American Heart Association Council on Epidemiology and Prevention, the Stroke Council, and the Functional Genomics and Translational Biology Interdisciplinary Working Group. Circulation 115: 2878-2901. doi:10.1161/CIRCULATIONAHA.107.183679. PubMed: 17515457. 17515457

[15] Sampietro ML, Trompet S, Verschuren JJ, Talens RP, Deelen J et al. (2011) A genome-wide association study identifies a region at chromosome 12 as a potential susceptibility locus for restenosis after percutaneous coronary intervention. Hum Mol Genet 20: 4748-4757. doi:10.1093/hmg/ddr389. PubMed: 21878436. 21878436 PMC3209827

[16] Langeveld B, Roks AJ, Tio RA, Voors AA, Zijlstra F, et al. (2005) Renin–angiotensin system intervention to prevent in-stent restenosis: an unclosed chapter. J 17 Cardiovasc Pharmacol 45:88–98 10.1097/00005344-200501000-00015 15613985

[17] Rakugi H, Wang DS, Dzau VJ, Pratt RE (1994) Potential importance of tissue angiotensin-converting enzyme inhibition in preventing neointima formation. Circulation 90: 449-455. doi:10.1161/01.CIR.90.1.449. PubMed: 7517799. 7517799

[18] Prisco D, Fatini C, Battaglini B, Gensini F, Fedi S et al. (2000) Angiotensin converting enzyme DD genotype affects the changes of plasma plasminogen activator inhibitor-1 activity after primary percutaneous transluminal coronary angioplasty in acute myocardial infarction patients. Int J Clin Lab Res 30: 179–185. doi:10.1007/s005990070004. PubMed: 11289708. 11289708

[19] Oike Y, Hata A, Ogata Y, Numata Y, Shido K et al. (1995) Angiotensin converting enzyme as a genetic risk factor for coronary artery spasm. Implication in the pathogenesis of myocardial infarction. J Clin Invest 96: 2975–2979. doi:10.1172/JCI118369. PubMed: 8675669. 8675669 PMC186009

[20] Bonithon-Kopp C, Ducimetière P, Touboul PJ, Fève JM, Billaud E et al. (1994) Plasma angiotensin converting enzyme activity and carotid wall thickening. Circulation 89: 952–954. doi:10.1161/01.CIR.89.3.952. PubMed: 8124834. 8124834

[21] Rakugi H, Kim DK, Krieger JE, Wang DS, Dzau VJ et al. (1994) Induction of angiotensin converting enzyme in the neointima after vascular injury. Possible role in restenosis. J Clin Invest 93: 339-346. doi:10.1172/JCI116965. PubMed: 8282805. 8282805 PMC293775

[22] Daemen MJAP, Lombardi DM, Bosman FT, Schwartz SM (1991) Angiotensin II induces smooth muscle cell proliferation in the normal and injured rat arterial wall. Circ Res 68: 450 - 456. doi:10.1161/01.RES.68.2.450. PubMed: 1991349. 1991349

[23] Agerholm-Larsen B, Nordestgaard BG, Tybjaerg-Hansen A (2000) ACE gene polymorphism in cardiovascular disease: Meta-analyses of small and large studies in whites. Arterioscler Thromb Vasc Biol 20: 484-492. doi:10.1161/01.ATV.20.2.484. PubMed: 10669647. 10669647

[24] Kammerer CM, Gouin N, Samollow PB, VandeBerg JF, Hixson JE et al. (2004) Two quantitative trait loci affect ACE activities in Mexican-Americans. Hypertension 43: 466-470. doi:10.1161/01.HYP.0000111830.36999.94. PubMed: 14707162. 14707162

[25] Ribichini F, Steffenino G, Dellavalle A, Matullo G, Colajanni E et al. (1998) Plasma activity and insertion/deletion polymorphism of angiotensin I-converting enzyme: A major risk factor and a marker of risk for coronary stent restenosis. Circulation 97: 147-154. doi:10.1161/01.CIR.97.2.147. PubMed: 9445166. 9445166

[26] Ohishi M, Fujii K, Minamino T, Higaki J, Kamitani A et al. (1993) A potent genetic risk factor for restenosis. Nat Genet 5: 324-325. doi:10.1038/ng1293-324. PubMed: 8298638. 8298638

[27] Nobuyoshi M, Kimura T, Nosaka H, Mioka S, Ueno K et al. (1988) Restenosis after successful percutaneous transluminal coronary angioplasty: serial angiographic follow-up of 229 patients. J Am Coll Cardiol 12: 616-623. doi:10.1016/S0735-1097(88)80046-9. PubMed: 2969925. 2969925

[28] Serruys PW, Luijten HE, Beatt KJ, Geuskens R, de Feyter PJ, et al. (1988) Incidence of restenosis after successful coronary angioplasty: a time-related phenomenon: a quantitative angiographic study in 342 consecutive patients at 1, 2, months. Circulation 77:361-71 10.1161/01.cir.77.2.361 2962786

[29] Tantai J, Shen Y, Zhao H (2013) Quantitative assessment of the influence of common variations on 6p21 and lung cancer risk. Tumour Biol: ([MedlinePgn:]) doi:10.1007/s13277-013-1094-3. PubMed: 23959479. 23959479

[30] Huang T, Hong J, Lin W, Yang Q, Ni K et al. (2013) Assessing Interactions between Common Genetic Variant on 2q35 and Hormone Receptor Status with Breast Cancer Risk: Evidence Based on 26. Studies - PLOS ONE 8: e69056. doi:10.1371/journal.pone.0069056. 23976942 PMC3745398

[31] Cochran WG (1954) The combination of estimates from different experiments. Biometrics 10: 101–129. doi:10.2307/3001666.

[32] Higgins JP, Thompson SG (2002) Quantifying heterogeneity in a meta-analysis. Stat Med 21: 1539–1558. doi:10.1002/sim.1186. PubMed: 12111919. 12111919

[33] DerSimonian R, Laird N (1986) Meta-analysis in clinical trials. Control Clin Trials 7: 177–188. doi:10.1016/0197-2456(86)90046-2. PubMed: 3802833. 3802833

[34] Mantel N, Haenszel W (1959) Statistical aspects of the analysis of data from retrospective studies of disease. J Natl Cancer Inst 22: 719–748. PubMed: 13655060. 13655060

[35] Zintzaras E, Lau J (2008) Synthesis of genetic association studies for pertinent gene-disease associations requires appropriate methodological and statistical approaches. J Clin Epidemiol 61: 634–645. doi:10.1016/j.jclinepi.2007.12.011. PubMed: 18538260. 18538260

[36] Begg CB, Mazumdar M (1994) Operating characteristics of a rank correlation test for publication bias. Biometrics 50: 1088–1101. doi:10.2307/2533446. PubMed: 7786990. 7786990

[37] Egger M, Davey Smith G, Schneider M, Minder C (1997) Bias in meta-analysis detected by a simple, graphical test. BMJ 315: 629–634. doi:10.1136/bmj.315.7109.629. PubMed: 9310563. 9310563 PMC2127453

[38] Beohar N, Damaraju S, Prather A, Yu QT, Raizner A et al. (1995) Angiotensin-I converting enzyme genotype DD is a risk factor for coronary artery disease. J Investig Med 43: 275-280. PubMed: 7614074. 7614074

[39] van Bockxmeer FM, Mamotte CD, Gibbons FA, Burke V, Taylor RR (1995) Angiotensin-converting enzyme and apolipoprotein E genotypes and restenosis after coronary angioplasty. Circulation 92: 2066-2071. doi:10.1161/01.CIR.92.8.2066. PubMed: 7554183. 7554183

[40] Kamitani A, Rakugi H, Higaki J, Ohishi M, Shi SJ et al. (1995) Enhanced predictability of myocardial infarction in Japanese by combined genotype analysis. Hypertension 25: 950-953. doi:10.1161/01.HYP.25.5.950. PubMed: 7737732. 7737732

[41] Kaski JC, Zhang Y, Calvino R, Vasquez › Rodriquez JM Castro>Beiras A, et al. (1996) Angiotensin-converting enzyme insertion/deletion polymorphism and restenosis after coronary angioplasty in unstable angina pectoris. Am J Cardiol 77:875-7 10.1016/s0002-9149(97)89187-4 8623745

[42] Tsukada K, Ishimitsu T, Tsuchiya N, Horinaka S, Matsuoka H (1997) Angiotensin-converting enzyme gene polymorphism and cardiovascular endocrine system in coronary angiography patients. Jpn Heart J 38: 799-810. doi:10.1536/ihj.38.799. PubMed: 9486933. 9486933

[43] Hamon M, Amant C, Bauters C, Richard F, Helbecque N et al. (1998) Dual determination of angiotensin-converting enzyme and angiotensin-II type 1 receptor genotypes as predictors of restenosis after coronary angioplasty. Am J Cardiol 81: 79-81. doi:10.1016/S0002-9149(97)00852-7. PubMed: 9462611. 9462611

[44] Yoshida M, Iwai N, Ohmichi N, Izumi M, Nakamura Y et al. (1999) D allele of the angiotensin-converting enzyme gene is a risk factor for secondary cardiac events after myocardial infarction. Int J Cardiol 70: 119-125. doi:10.1016/S0167-5273(99)00064-9. PubMed: 10454299. 10454299

[45] Okamura A, Ohishi M, Rakugi H, Katsuya T, Yanagitani Y et al. (1999) Pharmacogenetic analysis of the effect of angiotensin-converting enzyme inhibitor on restenosis after percutaneous transluminal coronary angioplasty. Angiology 50: 811-822. doi:10.1177/000331979905001005. PubMed: 10535720. 10535720

[46] Völzke H, Hertwig S, Rettig R, Motz W (2000) The angiotensinogen gene 235T variant is associated with an increased risk of restenosis after percutaneous transluminal coronary angioplasty. Clin Sci (Lond) 99: 19-25. doi:10.1042/CS19990277. PubMed: 10887054. 10887054

[47] Zee RYL, Fernandez-Ortiz A, Macaya C, Pintor E, Lindpaintner K et al. (2001) ACE D/I polymorphism and incidence of post-PTCA restenosis: a prospective, angiography-based evaluation. Hypertension 37: 851-855. doi:10.1161/01.HYP.37.3.851. PubMed: 11244007. 11244007

[48] Samani NJ, Martin DS, Brack M, Cullen J, Chauhan A et al. (1995) Insertion/deletion polymorphism in the angiotensin-converting enzyme gene and risk of restenosis after coronary angioplasty. Lancet 345: 1013-1016. doi:10.1016/S0140-6736(95)90756-4. PubMed: 7723497. 7723497

[49] Amant C, Bauters C, Bodart JC, Lablanche JM, Grollier G et al. (1997) D allele of the angiotensin I-converting enzyme is a major risk factor for restenosis after coronary stenting. Circulation 96: 56-60. doi:10.1161/01.CIR.96.1.56. PubMed: 9236417. 9236417

[50] Gensini F, Battaglini B, Fatini C, Guazzelli R, Falai M et al. (1999) Polimorfismo I/D del gene ACE e A1166C del gene AT1R quali fattori dirischio di restenosi dopo angioplastica coronarica. Minerva Cardioangiol 47: 516 PubMed: 10670180. 10670180

[51] Guarda E, Fajuri A, Marchant E, Martínez A, Jalil J et al. (1999) D/D genotype of the gene for angiotensin converting enzyme as a risk factor for post-stent coronary restenosis. Rev Esp Cardiol 52: 475-480. doi:10.1016/S0300-8932(99)74954-7. PubMed: 10439670. 10439670

[52] Gürlek A, Güleç S, Karabulut H, Bokesoy I, Tutar E et al. (2000) Relation between the insertion/deletion polymorphism of the angiotensin I converting enzyme gene and restenosis after coronary stenting. J Cardiovasc Risk 7: 403-407. PubMed: 11155292. 11155292 10.1177/204748730000700602

[53] Koch W, Kastrati A, Mehilli J, Böttiger C, van Beckerlath N et al. (2000) Insertion/Deletion polymorphism of the angiotensin I-converting enzyme gene is not associated with restenosis after coronary stent placement. Circulation 102: 197-202. doi:10.1161/01.CIR.102.2.197. PubMed: 10889131. 10889131

[54] Jørgensen E, Kelbaek H, Helqvist S, Jensen GV, Saunamäki K et al. (2001) Predictors of coronary in-stent restenosis: importance of angiotensin-converting enzyme gene polymorphism and treatment with angiotensin-converting enzyme inhibitors. J Am Coll Cardiol 38: 1434-1439. doi:10.1016/S0735-1097(01)01580-7. PubMed: 11691520. 11691520

[55] Taniguchi I, Yamazaki T, Wagatsuma K, Kurusu T, Shimazu Y et al. (2001) The DD genotype of angiotensin converting enzyme polymorphism is a risk factor for coronary artery disease and coronary stent restenosis in Japanese patients. Jpn Circ J 65: 897-900. doi:10.1253/jcj.65.897. PubMed: 11665795. 11665795

[56] Ferrari M, Mudra H, Grip L, Voudris V, Schächinger V et al. (2002) Angiotensin-converting enzyme insertion/deletion polymorphism does not influence the restenosis rate after coronary stent implantation. Cardiology 97: 29-36. doi:10.1159/000047416. PubMed: 11893827. 11893827

[57] Ryu SK, Cho EY, Park HY, Im EK, Jang YS et al. (2002) Renin-angiotensin-aldosterone system (RAAS) gene polymorphism as a risk factor of coronary in-stent restenosis. Yonsei Med J 43: 461-472. PubMed: 12205735. 12205735 10.3349/ymj.2002.43.4.461

[58] Gomma AH, Elrayess MA, Knight CJ, Hawe E, Fox KM et al. (2002) The endothelial nitric oxide synthase (Glu298Asp and -786T>C) gene polymorphisms are associated with coronary in-stent restenosis. Eur Heart J 23: 1955-1962. doi:10.1053/euhj.2002.3400. PubMed: 12473258. 12473258

[59] Qu X, Zhao J, Liu P, Song L, Zhao K et al. (2002) Relationship between angiotensin-1 converting enzyme gene polymorphism and in-stent restenosis. Zhonghua Yi Xue Za Zhi 82: 474-6.53. 12133519

[60] Okumura K, Sone T, Kondo J, Tsuboi H, Mukawa H et al. (2002) Quinapril prevents restenosis after coronary stenting in patients with angiotensin-converting enzyme D allele. Circ J 66: 311-316. doi:10.1253/circj.66.311. PubMed: 11954942. 11954942

[61] Ribichini F, Wijns W, Ferrero V, Matullo G, Camilla T et al. (2003) Effect of angiotensin-converting enzyme inhibition on restenosis after coronary stenting. Am J Cardiol 91: 154-8.36 PubMed: 12521626. 12521626 10.1016/s0002-9149(02)03101-6

[62] Guo F, Chen LJ, Xie ZJ, Liu W, Li JY et al. (2005) Association of renin-angiotensin system gene polymorphisms and the incidence of in-stent restenosis. Zhongguo You Sheng Yu Yi Chuan za Zhi 13: 18-20.

[63] Wang H, Yang ZJ, Ma GS, Zhu TB, Yin H et al. (2005) Polymorphism of the angiotensin-converting enzyme gene associated with incidence of restenosis following coronary stent placement. Zhongguo Lin Chuang Kang FU 9: 33-35.

[64] Guneri S, Baris N, Aytekin D, Akdeniz B, Pekel N et al. (2005) The relationship between angiotensin converting enzyme gene polymorphism, coronary artery disease, and stent restenosis: the role of angiotensin converting enzyme inhibitors in stent restenosis in patients with diabetes mellitus. Int Heart J 46: 889-897. doi:10.1536/ihj.46.889. PubMed: 16272779. 16272779

[65] Wang SJ, Ge JB, Sun AJ, Yuan ZG, Zhang HQ et al. (2005) Relationship between the polymorphism of angiotensin I converting enzyme gene and instent restenosis after PTCA with stent planting. Zhongguo Lin Chuang Yi Xue 12: 386-388.

[66] Ujiie Y, Hirosaka A, Mitsugi M, Ohwada T, Igarashi M et al. (2006) Effects of angiotensin-converting enzyme inhibitors or an angiotensin receptor blocker in combination with aspirin and cilostazol on in-stent restenosis. Int Heart J 47: 173-184. doi:10.1536/ihj.47.173. PubMed: 16607045. 16607045

[67] Gao CX, Chang TX, Li YH, Wang SS, Liu W et al. (2006) Association between gene polymorphism of renin-angiotensin system and in-stent restenosis after percutaneous transluminal coronary angioplasty. Nanjing Yi Ke da Xue Xue Bao 26: 351-355.

[68] Wijpkema JS, van Haelst PL, Monraats PS, Bruinenberg M, Zwinderman AH et al. (2006) Restenosis after percutaneous coronary intervention is associated with the angiotensin-II type-1 receptor 1166A/C polymorphism but not with polymorphisms of angiotensin-converting enzyme, angiotensin-II receptor, angiotensinogen or heme oxygenase-1. Pharmacogenet Genomics 16: 331-337. doi:10.1097/01.fpc.0000205001.07054.fa. PubMed: 16609364. 16609364

[69] Lv D, He ZF (2012) Association study of angiotensin converting enzyme gene I/D polymorphism and restenosis after coronary stent placement. Zhongguo Fen Zi Xin Zang Bing Xue za Zhi 12: 76-78.

[70] Fischman DL, Leon MB, Baim DS, Schatz RA, Savage MP et al. (1994) A randomized comparison of coronary-stent placement and balloon angioplasty in the treatment of coronary artery disease. Stent Restenosis Study Investigators. N Engl J Med 331: 496-501. doi:10.1056/NEJM199408253310802. PubMed: 8041414. 8041414

[71] Serruys PW, van Hout B, Bonnier H, Legrand V, Garcia E et al. (1998) Randomised comparison of implantation of heparin-coated stents with balloon angioplasty in selected patients with coronary artery disease (Benestent II). Lancet 352: 673-681. doi:10.1016/S0140-6736(97)11128-X. PubMed: 9728982. 9728982

[72] Ross R (1999) Atherosclerosis--an inflammatory disease. N Engl J Med 340: 115-126. doi:10.1056/NEJM199901143400207. PubMed: 9887164. 9887164

[73] Lee MS, David EM, Makkar RR, Wilentz JR (2004) Molecular and cellular basis of restenosis after percutaneous coronary intervention: the intertwining roles of platelets, leukocytes, and the coagulation-fibrinolysis system. J Pathol 203: 861-870. doi:10.1002/path.1598. PubMed: 15258987. 15258987

[74] Rigat B, Hubert C, Alhenc-Gelas F, Cambien F, Corvol P et al. (1990) An insertion/deletion polymorphism in the angiotensin I-converting enzyme gene accounting for half the variance of serum enzyme levels. J Clin Invest 86: 1343–1346. doi:10.1172/JCI114844. PubMed: 1976655. 1976655 PMC296868

[75] Danser AH, Schalekamp MA, Bax WA, van den Brink AM, Saxena PR et al. (1995) Angiotensin-converting enzyme in the human heart: Effects of the deletion/insertion polymorphism. Circulation 92: 1387-1388. doi:10.1161/01.CIR.92.6.1387. PubMed: 7664416. 7664416

[76] Castellano M, Muiesan ML, Rizzoni D, Beschi M, Pasini G et al. (1995) Angiotensin-converting enzyme I/D polymorphism and arterial wall thickness in a general population: the Vobarno Study. Circulation 91: 2721-2724. doi:10.1161/01.CIR.91.11.2721. PubMed: 7758176. 7758176

[77] Powell JS, Clozel JP, Müller RKM, Kuhn H, Hefti F et al. (1989) Inhibitors of angiotensin-converting enzyme prevent myointimal proliferation after vascular injury. Science 245: 186-188. doi:10.1126/science.2526370. PubMed: 2526370. 2526370

[78] Daemon MJAP, Lombardi DM, Bosman FT, Schwartz SM (1991) Angiotensin II induces smooth muscle cell proliferation in the normal and injured rat arterial wall. Circ Res 68: 451-456. 10.1161/01.res.68.2.450 1991349

[79] Faxon DP (1995) Effect of high dose angiotensin-converting enzyme inhibition on restenosis: Final results of the MARCATOR study, a multicenter, double-blind, placebo-controlled trial of cilazapril. J Am Coll Cardiol 25: 362-369. doi:10.1016/0735-1097(95)92960-D. PubMed: 7829789. 7829789

[80] The Multicenter European Research Trial With Cilazapril After Angioplasty to Prevent Transluminal Coronary Obstruction and Restenosis (MERCATOR) Study Group (1992) Does the new angiotensin converting enzyme inhibitor cilazapril prevent restenosis after percutaneous transluminal coronary angioplasty? Circulation 86:100-110 8357332

[81] Desmet W, Vrolix M, Scheerder ID, Lierde JV, Willems JL et al. (1994) Angiotensin-converting enzyme inhibition with fosinopril sodium in the prevention of restenosis after coronary angioplasty. Circulation 89: 385-392. doi:10.1161/01.CIR.89.1.385. PubMed: 8281674. 8281674

[82] Daemen MJ, Lombardi DM, Bosman FT, Schwartz SM (1991) Angiotensin II induces smooth muscle cell proliferation in the normal and injured rat arterial wall. Circ Res 68: 450-456. doi:10.1161/01.RES.68.2.450. PubMed: 1991349. 1991349

[83] Farhy RD, Carretero OA, Ho KL, Scicli AG (1993) Role of kinins and nitric oxide in the effects of angiotensin converting enzyme inhibitors on neointima formation. Circ Res 72: 1202-1210. doi:10.1161/01.RES.72.6.1202. PubMed: 7684331. 7684331

[84] Morishita R, Gibbons GH, Ellison KE, Lee W, Zhang L et al. (1994) Evidence for direct local effect of angiotensin in vascular hypertrophy. In vivo gene transfer of angiotensin converting enzyme. J Clin Invest 94: 978-984. doi:10.1172/JCI117464. PubMed: 8083382. 8083382 PMC295142

[85] Kakuta T, Currier JW, Haudenschild CC, Ryan TJ, Faxon DP (1994) Differences in compensatory vessel enlargement, not intimal formation, account for restenosis after angioplasty in the hypercholesterolemic rabbit model. Circulation 89: 2809-2815. doi:10.1161/01.CIR.89.6.2809. PubMed: 8205695. 8205695

[86] Lafont A, Guzman LA, Whitlow PL, Goormastic M, Cornhill JF et al. (1995) Restenosis after experimental angioplasty. Intimal, medial, and adventitial changes associated with constrictive remodeling. Circ Res 76: 996-1002. doi:10.1161/01.RES.76.6.996. PubMed: 7758171. 7758171

[87] Mintz GS, Popma JJ, Pichard AD, Kent KM, Satler LF et al. (1996) Arterial remodeling after coronary angioplasty: a serial intravascular ultrasound study. Circulation 94: 35-43. doi:10.1161/01.CIR.94.1.35. PubMed: 8964115. 8964115

[88] Passaro A, Calzoni F, Volpato S, Nora ED, Pareschi PL et al. (2003) Effect of metabolic control on homocysteine levels in type 2 diabetic patients: a 3-year follow-up. J Intern Med 254: 264–271. doi:10.1046/j.1365-2796.2003.01184.x. PubMed: 12930236. 12930236

[89] Ma J, Stampfer MJ, Giovannucci E, Artigas C, Hunter DJ et al. (1997) Methylenetetrahydrofolate reductase polymorphism, dietary interactions, and risk of colorectal cancer. Cancer Res 57: 1098–1102. PubMed: 9067278. 9067278

