# Supplementary figures and images for: Influence of the Angiotensin Converting Enzyme Insertion or Deletion Genetic Variant and Coronary Restenosis Risk: Evidence Based on 11,193 Subjects
Source: PLoS One. 2013 Dec 13;8(12):e83415. doi: 10.1371/journal.pone.0083415 (PMC3862770; doi:10.1371/journal.pone.0083415)

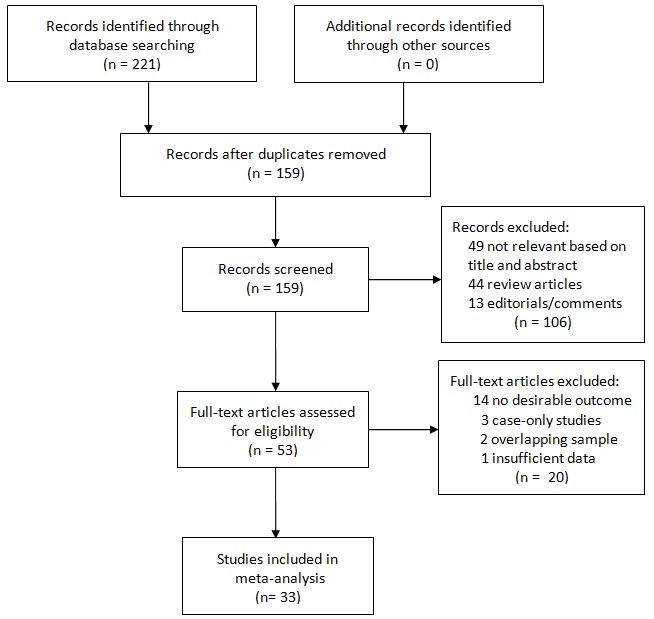

Supplement: Figure S1 — Flow chart of literature search for studies examining ACE I/D polymorphism and risk of restenosis after PTCA. (TIF) [file pone.0083415.s002.tif]

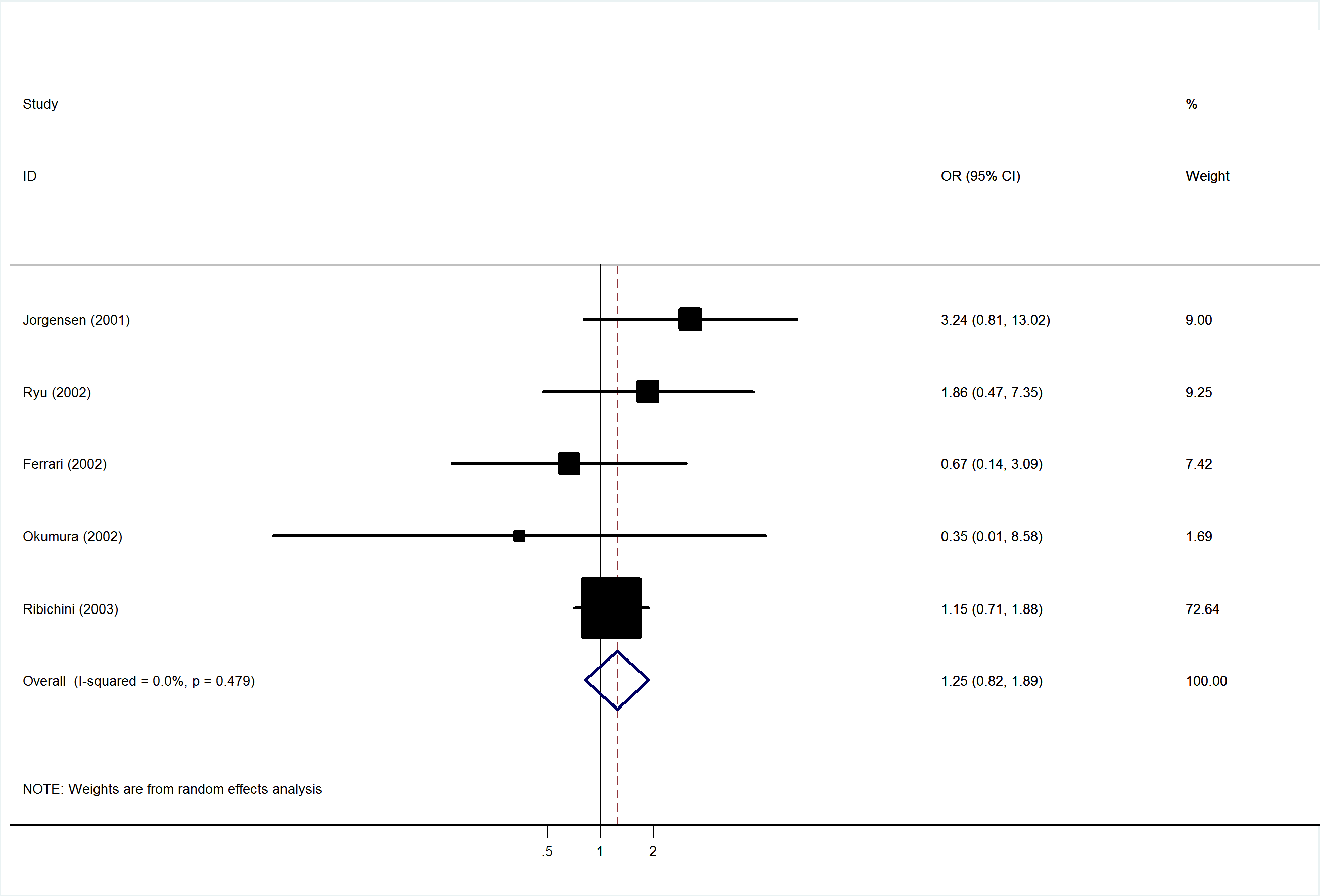

Supplement: Figure S2 — Association between ACE I/D polymorphism and restenosis risk after PTCA-stent by ACE inhibitors treatment. (TIF) [file pone.0083415.s003.tif]

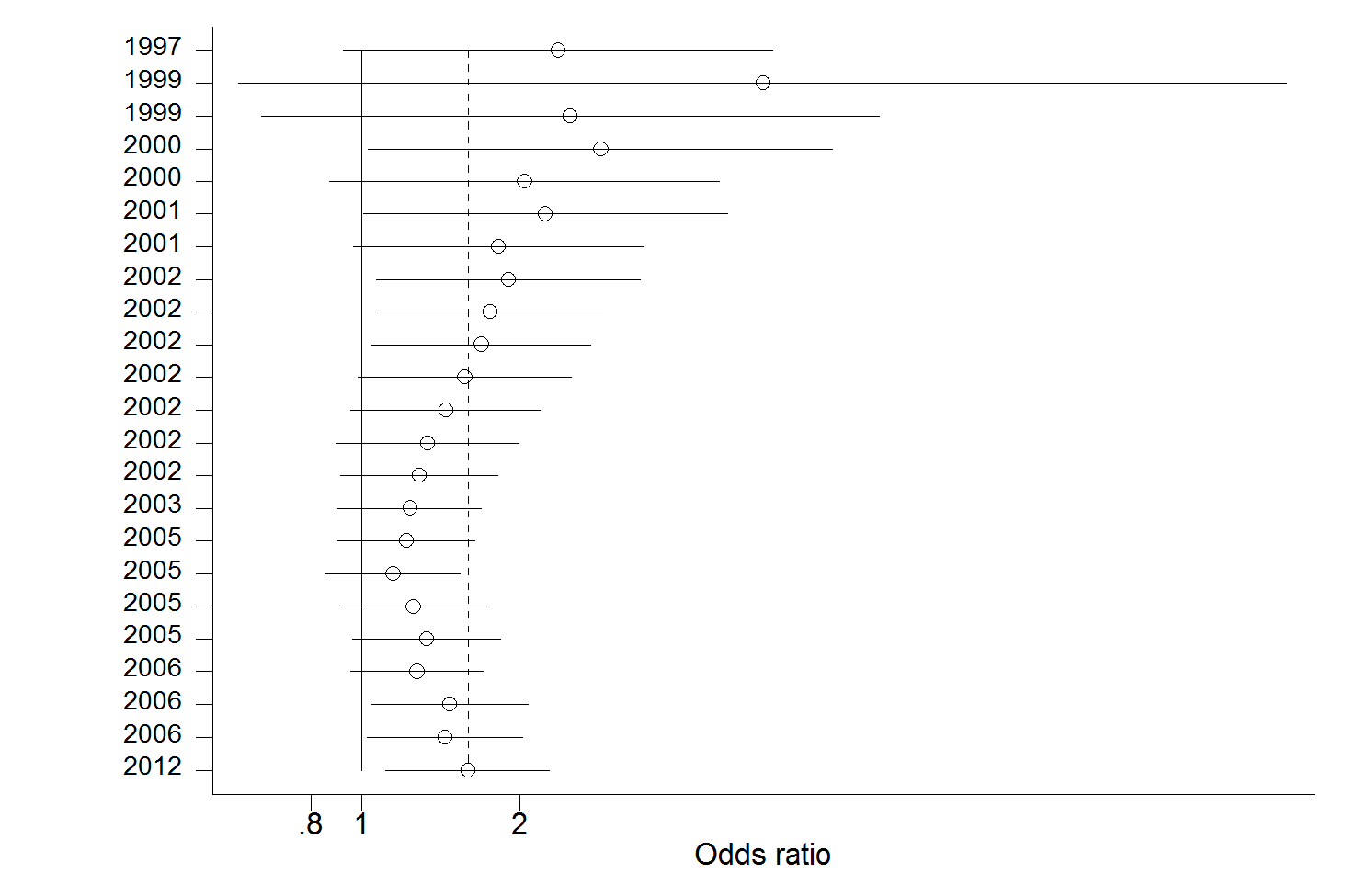

Supplement: Figure S3 — Cumulative meta-analysis for restenosis after PTCA-stent and ACE I/D polymorphism: the random effects pooled odds ratio (OR) with the corresponding 95% confidence interval (CI) at the end of each year-information step is shown. (TIF) [file pone.0083415.s004.tif]

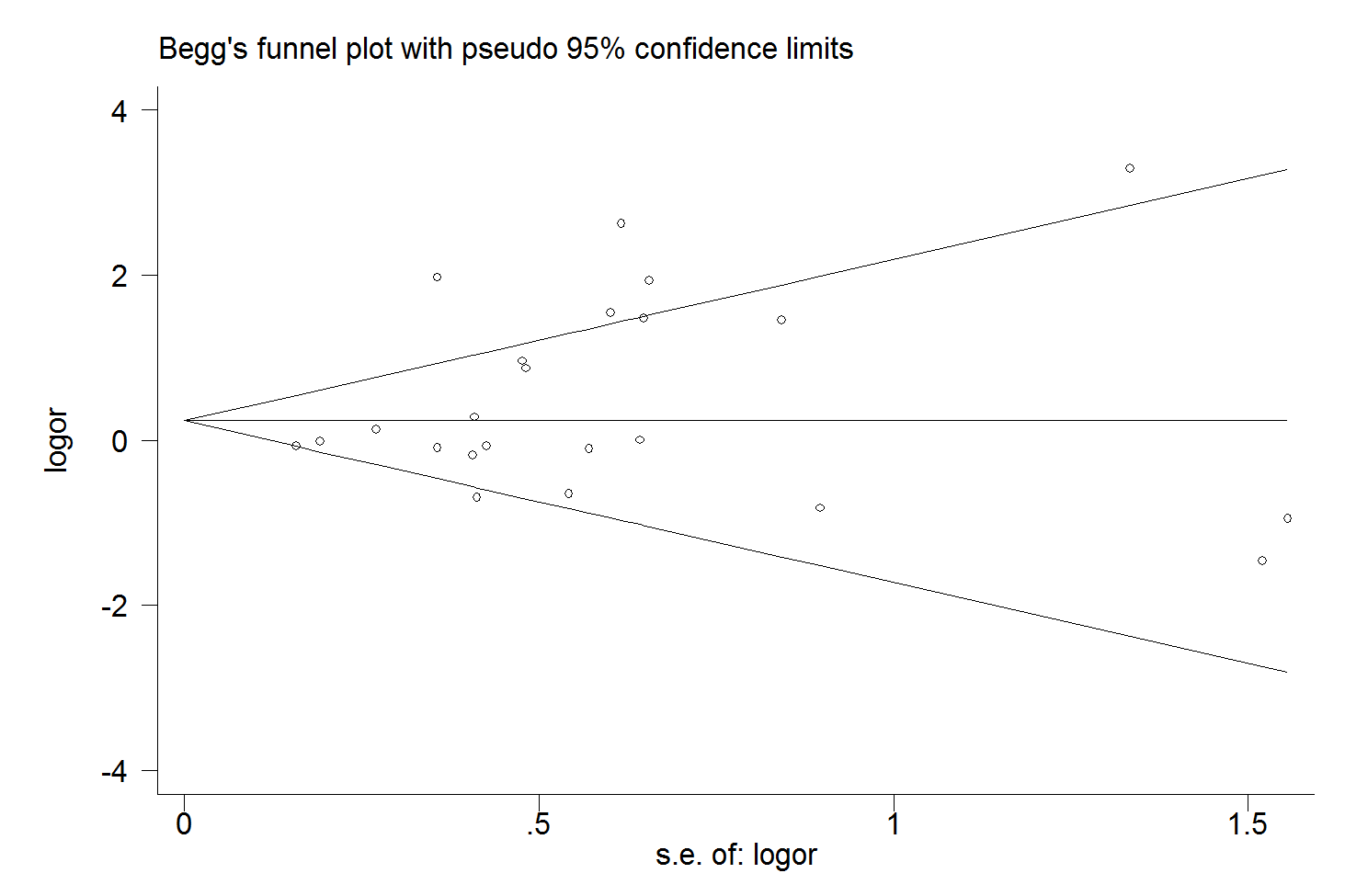

Supplement: Figure S4 — Begg’s funnel plot for publication bias in studies on ACE I/D polymorphism and restenosis after PTCA-stent. (TIF) [file pone.0083415.s005.tif]

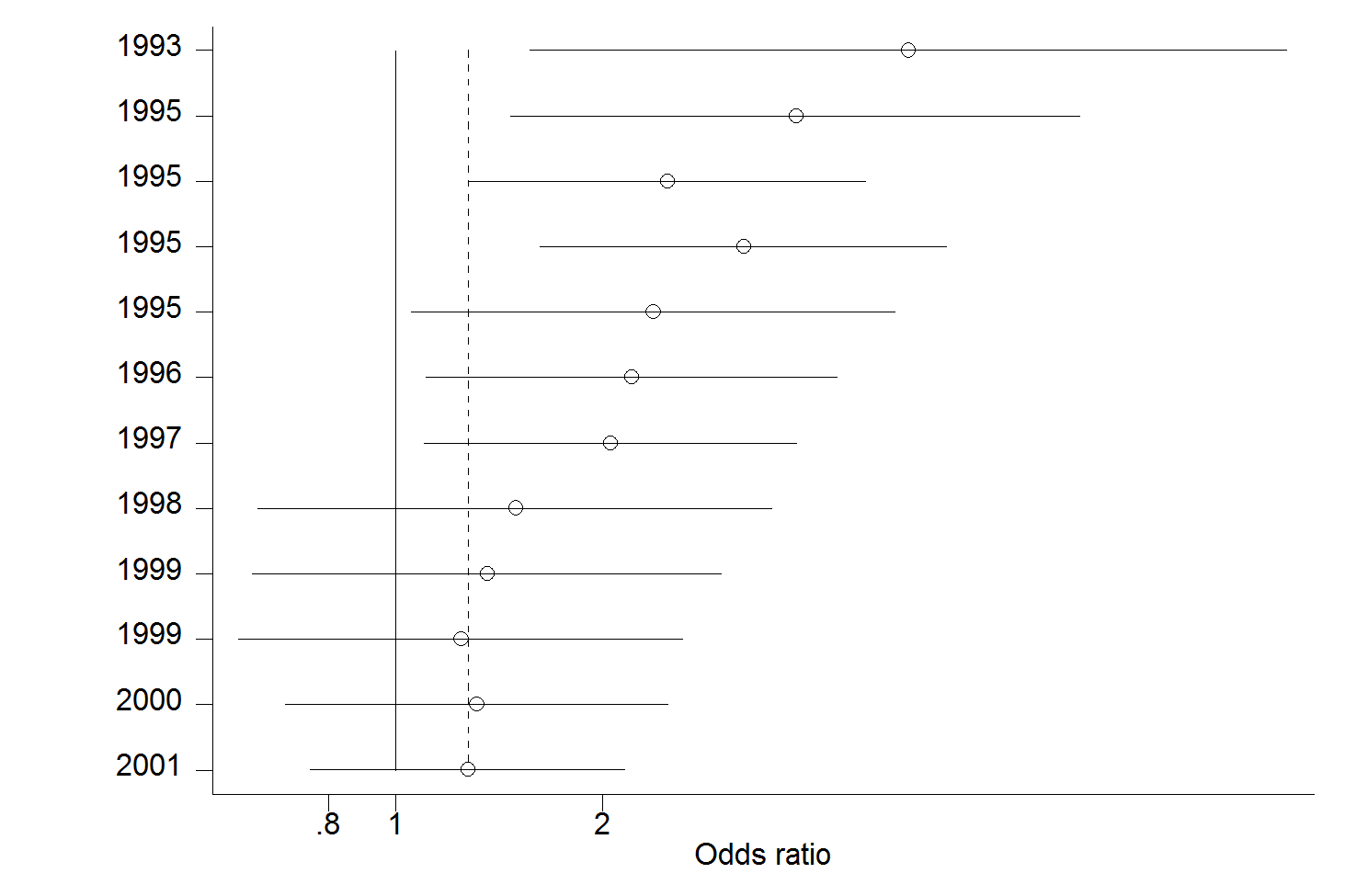

Supplement: Figure S5 — Cumulative meta-analysis for restenosis after PTCA-balloon and ACE I/D polymorphism. (TIF) [file pone.0083415.s006.tif]

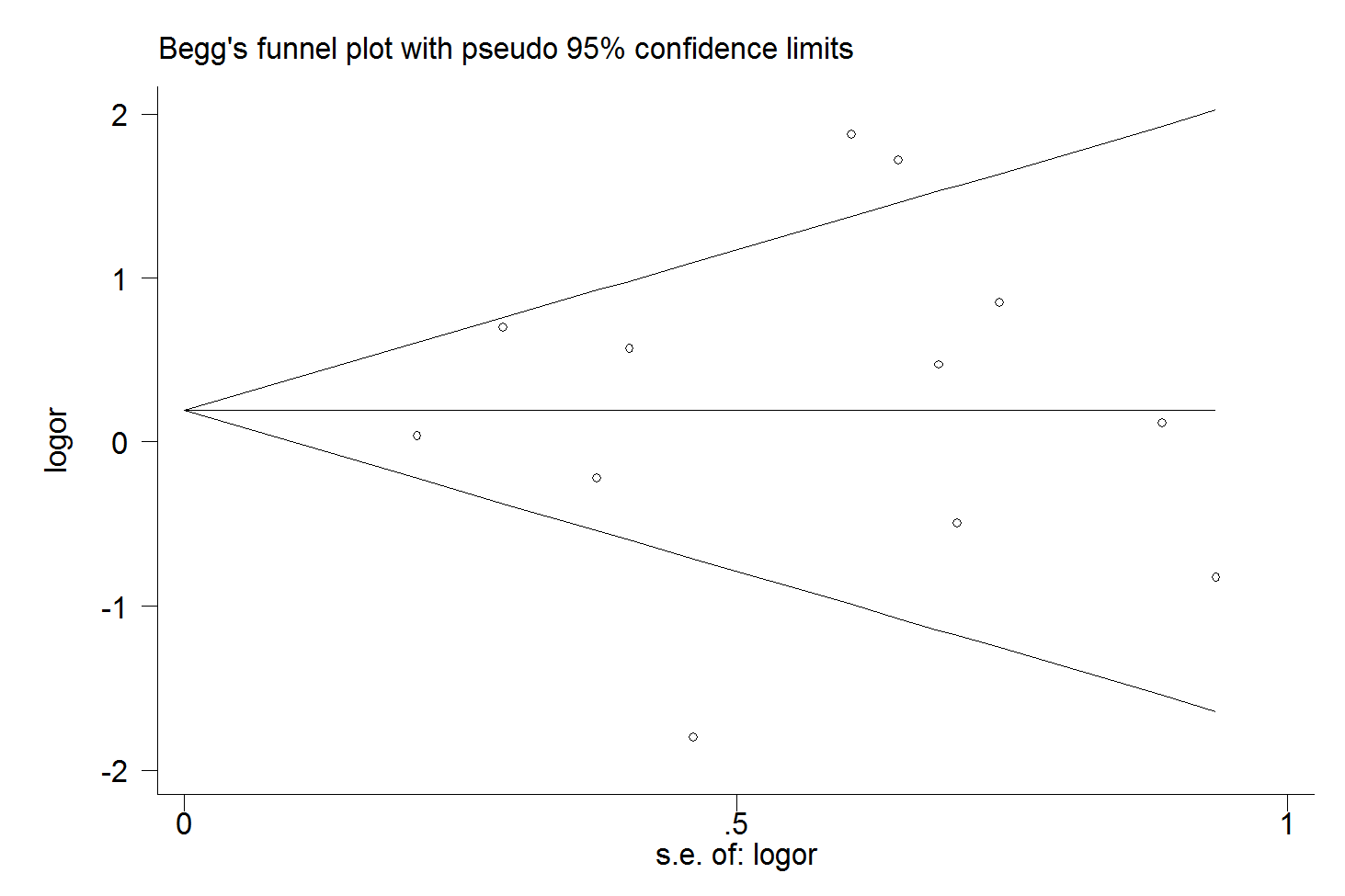

Supplement: Figure S6 — Begg’s funnel plot for publication bias in studies on ACE I/D polymorphism and restenosis after PTCA-balloon. (TIF) [file pone.0083415.s007.tif]
